# Transcriptional Regulation of Drought Response in *Arabidopsis* and Woody Plants

**DOI:** 10.3389/fpls.2020.572137

**Published:** 2021-01-08

**Authors:** Tao Yao, Jin Zhang, Meng Xie, Guoliang Yuan, Timothy J. Tschaplinski, Wellington Muchero, Jin-Gui Chen

**Affiliations:** ^1^Biosciences Division, Oak Ridge National Laboratory, Oak Ridge, TN, United States; ^2^Center for Bioenergy Innovation, Oak Ridge National Laboratory, Oak Ridge, TN, United States; ^3^State Key Laboratory of Subtropical Silviculture, School of Forestry and Biotechnology, Zhejiang A&F University, Hangzhou, China; ^4^Biology Department, Brookhaven National Laboratory, Upton, NY, United States

**Keywords:** *Populus*, *Arabidopsis*, transcription factor, transcriptional regulation, abscisic acid, drought response

## Abstract

Within the context of global warming, long-living plants such as perennial woody species endure adverse conditions. Among all of the abiotic stresses, drought stress is one of the most detrimental stresses that inhibit plant growth and productivity. Plants have evolved multiple mechanisms to respond to drought stress, among which transcriptional regulation is one of the key mechanisms. In this review, we summarize recent progress on the regulation of drought response by transcription factor (TF) families, which include abscisic acid (ABA)-dependent ABA-responsive element/ABRE-binding factors (ABRE/ABF), WRKY, and Nuclear Factor Y families, as well as ABA-independent AP2/ERF and NAC families, in the model plant *Arabidopsis*. We also review what is known in woody species, particularly *Populus*, due to its importance and relevance in economic and ecological processes. We discuss opportunities for a deeper understanding of drought response in woody plants with the development of high-throughput omics analyses and advanced genome editing techniques.

## Introduction

Plant growth and development are affected by environmental stimuli including biotic and abiotic stresses. Drought stress is one of the most deleterious abiotic stresses to plants caused by limited rainfall, rising temperatures, and insufficient water availability and has become an increasing concern because of global climate change (Yamaguchi-Shinozaki and Shinozaki, 2006; Nakashima et al., 2014; Gupta et al., 2020a). Drought stress causes a series of injuries in terms of plant physiological, biochemical, and metabolic impacts, which result in plant growth retardation, cell damage, and leads to loss of crop yield and quality (Joshi et al., 2016). The sessile nature of plants requires them to develop effective responsive mechanisms, including escape (i.e., accelerating flowering), avoidance (i.e., maintaining high internal water content), and tolerance (i.e., maintaining growth under low internal water content) to adapt to drought stress (Salehi-Lisar and Bakhshayeshan-Agdam, 2016; Gupta et al., 2020a).

In response to drought stress, stomatal closure is an early and rapid response to avoid water loss, although it also can lead to negative effects on photosynthesis (Raghavendra et al., 2010). Morphological changes, including wax biosynthesis on leaf surface and enhanced root growth are other strategies adopted by plants to reduce respiration and improve water uptake, respectively, (Aharoni et al., 2004; Meng et al., 2019). Secondly, plants produce functional proteins, such as dehydrins and late embryogenesis-abundant proteins (LEA), as well as wide spectrum of metabolites to relieve potential osmotic damage from drought stress. Thirdly, plants produce antioxidant enzymes to scavenge reactive oxygen species (ROS) and protect cells from oxidative damage induced by drought stress (Claeys and Inze, 2013). Noticeably, stomatal closure is through a complex membrane transporter system to quickly respond to drought stress (Raghavendra et al., 2010; Gong et al., 2020). Most of the other responses are typically under the control of transcriptional regulation in which transcription factors (TFs) play the pivotal roles (Takahashi et al., 2018).

Much of the progress of drought response studies has been made in the model plants *Arabidopsis thaliana* and rice (*Oryza sativa*) over the past decades (Shinozaki and Yamaguchi-Shinozaki, 2006; Joshi et al., 2016). As a stress hormone, abscisic acid (ABA) is abruptly synthesized in response to different stresses, including drought, enhancing plant drought tolerance through closing stomata and restraining plant growth (Zeevaart, 1980; Zhu, 2016). ABA is perceived by the PYR/PYL/RCAR receptors, which activate downstream TFs, such as ABA-responsive element (ABRE)-binding proteins (AREBs)/ABRE-binding factors (ABFs) by cascade kinase reactions. Activated ABA signaling enhances drought tolerance by inducing stress-responsive genes. However, expression of some drought-responsive genes is independent of ABA. For example, *RD29A* and *ERD1* can be induced by drought in an ABA-independent pathway. Their expression is controlled by other TFs families, such as APETALA2/ethylene-responsive factor (AP2/ERF), and NAM, ATAF1/2, and CUC2 (NAC) (Takahashi et al., 2018).

Similar to *Arabidopsis* and rice, woody plant species are also negatively affected by drought, including reduced plant growth, inhibited wood formation, and increased susceptibility to pathogens (Fernàndez-Martínez et al., 2013; Yin et al., 2014; Polle et al., 2019). With the approaches of genetics and omics, studies on response of woody plants have identified many important TFs, and the transcriptional regulation of drought response is emerging (Estravis-Barcala et al., 2019). In this review, we will provide an update on the recent progress of the role of TFs in drought response in the model plant, *Arabidopsis*, and highlight findings in the woody genus, *Populus*.

## ABA-Dependent Drought Response Pathway

The ABA signaling pathway consists of receptor RCAR/PYR/PYLs, protein phosphatase PP2C, kinase SnRK2s (SnRK2.2, SnRK2.3, and SnRK2.6), and the targeting substrates (Umezawa et al., 2010; Guo et al., 2011). Once bound and activated by ABA, PYR/PYL/RCARs form a trimeric complex with PP2Cs, which inhibits the phosphatase activity of PP2Cs. SnRK2s are then released from the association with PP2Cs. Released SnRK2s can be activated by autophosphorylation, and, in turn, phosphorylate the downstream TFs and ion channel proteins (Fujita et al., 2009; Ma et al., 2009; Park et al., 2009; Umezawa et al., 2009). Among SnRK2 targets, AREBs/ABFs are the downstream TFs in the ABA signaling pathway. Some other TF families, such as WRKY, MYB, and NF-Ys, are also involved in drought response and adaption (Singh and Laxmi, 2015).

### AREBs/ABFs

**FIGURE 1:**
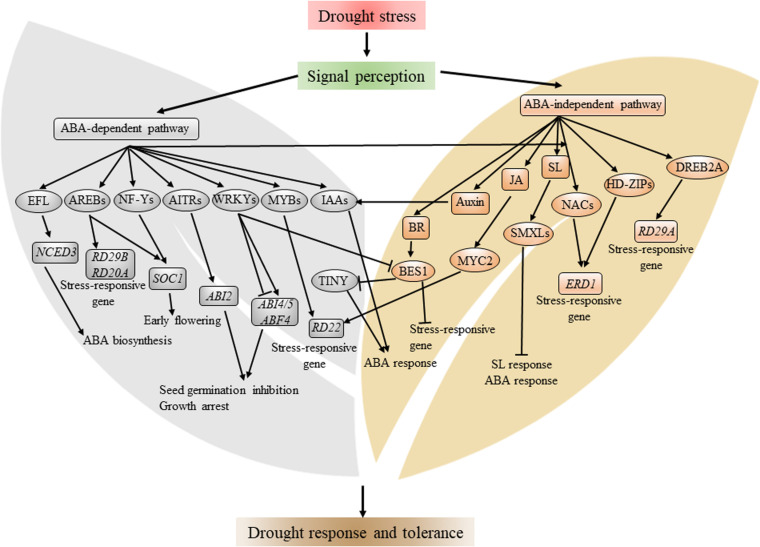
Schematic representation of major transcription factors and transcriptional network involved in drought stress response. Drought stress is perceived by plant in an unclear mechanism, which induces the biosynthesis of ABA. ABA signaling, in turn, activates several TF families, including AREB, NF-Y, WRKY, and MYB. There are ABA-independent TFs involved in drought stress, such as DREB2A, HD-ZIP, MYC2, and several NACs. These TFs, individually or cooperatively, regulate plant growth and development, metabolism, and expression of stress-inducible genes, such as *RD22* and *ERD1*. Arrows and bars indicate activation and inhibition of the expression of downstream genes, respectively. Components in the gray background denote the ABA-dependent pathway, and components in brown background denote the ABA-independent pathway.

Gene expression microarray and RNA-seq approaches have been used extensively to identify drought-responsive genes. Through the analysis of *cis*-acting promoter elements, 8-bp-long ABRE, PyACGTGGC, was identified in the promoter in 82% of dehydration-responsive genes in *Arabidopsis* (Maruyama et al., 2012). ABRE are bound by AREBs/ABFs, which belong to the bZIP TF family. Four members of AREBs/ABFs (AREB1, AREB2, ABF3, and ABF1), whose expression are induced by both dehydration and ABA treatment, were reported to regulate drought response through the ABA-dependent pathway. The quadruple mutant of *areb1 areb2 abf3 abf1* showed a drought-sensitive phenotype and reduced sensitivity to ABA (Yoshida et al., 2010, 2015). ENHANCED EM LEVEL (EFL), belonging to bZIP TF family, formed a protein complex with GIGANTEA (GI) to regulate diurnal ABA biosynthesis contributing to drought tolerance (Baek et al., 2020a). Noticeably, an ABRE is required to co-locate with other copies of ABRE or coupling elements (CEs) to activate ABA-responsive genes (Figure 1). Since ABA regulates most of its target genes through AREBs/ABFs, the ABRE element is recognized as a key signature for drought-responsive genes regulated by the ABA-dependent pathway.

Recently, many ABA signaling components have been reported to be involved in drought response in woody plants. Overexpression of the ABA receptor, *PtPYRL1* or *PtPYRL5*, increased drought tolerance (Yu et al., 2017). Accordingly, *Arabidopsis* transgenic plants overexpressing *Populus PP2C* genes negatively regulated drought tolerance and showed enhanced water loss (Arshad and Mattsson, 2014; Chen et al., 2015). There are 14 putative AREB/ABF members encoded in the *Populus* genome. Eight of them were upregulated upon exogenous ABA treatment, whereas the other six members were downregulated (Ji et al., 2013). Transgenic plants overexpressing *PtrAREB3* showed a strong drought tolerance phenotype under drought conditions with compromised biomass production (Yu et al., 2019). *PeABF3*, isolated from *Populus euphratica*, was induced by dehydration and ABA treatment. Overexpression of *PeABF3* enhance drought tolerance by promoting ABA-induced stomatal closure (Yang Y. et al., 2020). Li et al. (2019) identified four *ABRE1* homologs in *P. trichocarpa*, three of which (*PtrAREB1-2*, *PtrAREB1-3*, and *PtrAREB1-4*) were induced by drought treatment. Knocking down of *PtrAREB1* showed reduced drought tolerance in transgenic poplar (Table 1). PtrAREB1-2 was reported to recruit HAT complex proteins, ADA2b and GCN5, to increase H3K9 acetylation and activate the expression of downstream genes, such as *PtrNACs* (Li et al., 2019). Besides AREB/ABFs, another bZIP TF PtabZIP1L contributed to drought tolerance by increasing lateral root formation and modulating the biosynthesis of the drought tolerance-related metabolites (Dash et al., 2017). In conclusion, ABA biosynthesis and the ABA signaling pathway play pivotal roles in drought stress response in woody plants, such as poplar.

**TABLE 1:** Summary of the transcription factors involved in drought response in woody plants.

Gene family	Gene symbol	Identified from species	Studied in species	Pathway	Description	References
AREB/ABF	*PtrAREB3*	*Populus trichocarpa*	*Populus tremula* × *tremuloides* “T89”	ABA responsive	Positive regulator	Yu et al., 2019
	*PtrAREB1-2*	*Populus trichocarpa*	*Populus trichocarpa*	Drought inducible	Positive regulator	Li et al., 2019
	*PeABF3*	*Populus euphratica*	*Populus tomentosa*	Mannitol, ABA, dehydration-inducible	Positive regulator	Yang Y. et al., 2020
WRKY	*PbrWRKY53*	*Pyrus betulaefolia*	Tobacco, *Pyrus ussuriensis*	ABA, drought inducible	Positive regulator	Liu Y. et al., 2019
NF-Y	*PtNF-YA9*	*Populus trichocarpa*	*Arabidopsis thaliana*	Mannitol, NaCl, ABA repressible	Positive regulator	Lian et al., 2018
	*PdNF-YB7*	*Populus nigra* × (*P. deltoides* × *P. nigra*)	*Arabidopsis thaliana*	PEG6000, ABA inducible	Positive regulator	Han et al., 2013
	*PdNF-YB21*	*Populus nigra* × (*P. deltoides* × *P. nigra*)	*Populus alba* × *P. glandulosa*	ABA, osmotic, drought inducible	Negative regulator	Zhou et al., 2020
MYB	*PtoMYB170*	*Populus tomentosa*	*Arabidopsis thaliana*	Unknown	Positive regulator	Xu et al., 2017
	*PtrMYB94*	*Populus trichocarpa*	*Arabidopsis thaliana*, *Populus tomentosa*	Dehydration, ABA inducible	Positive regulator	Fang et al., 2019
AP2/ERF	*PeDREB2L*	*Populus euphratica* Oliva	*Arabidopsis thaliana*	Dehydration, salt, ABA inducible	Positive regulator	Chen et al., 2011
	*PeDREB2a*	*Populus euphratica*	*Arabidopsis thaliana*	PEG, salt, cold inducible	Positive regulator	Zhou et al., 2012
	*PeSHN1*	*Populus* × *euramericana* “Neva”	*Populus alba* × *P. glandulosa*	Dehydration, ABA inducible	Positive regulator	Meng et al., 2019
	*MdERF38*	*Malus* × *domestic* “Gala”	*Malus* × *domestic* “Gala” and *Arabidopsis thaliana*	PEG inducible	Positive regulator	An et al., 2020
	*MdWRI4*	*Malus* × *domestic* “Gala”	*Malus* × *domestic* “Gala”	PEG, ABA, and NaCl-inducible	Positive regulator	Zhang et al., 2020
	*MdSHINE2*	*Malus* × *domestic* “Gala”	*Arabidopsis thaliana*	PEG, ABA, GA and NaCl-inducible	Positive regulator	Zhang Y.-L. et al., 2019
NAC	*PeNAC036*	*Populus euphratica*	*Arabidopsis thaliana*	Drought, salt inducible	Positive regulator	Lu et al., 2018
	*PeNAC034*	*Populus euphratica*	*Arabidopsis thaliana*	Drought, salt repressible	Negative regulator	Lu et al., 2018
	*PeNAC045*	*Populus euphratica*	*Populus tomentosa*	Drought, salt repressible	Negative regulator	Lu et al., 2018
	*PtrNAC006*	*Populus trichocarpa*	*Populus trichocarpa*	Drought inducible	Positive regulator	Li et al., 2019
	*PtrNAC007*	*Populus trichocarpa*	*Populus trichocarpa*	Drought inducible	Positive regulator	Li et al., 2019
	*PtrNAC120*	*Populus trichocarpa*	*Populus trichocarpa*	Drought inducible	Positive regulator	Li et al., 2019
	*VaNAC26*	*Vitis amurensis*	*Arabidopsis thaliana*	Cold, drought, salt inducible	Positive regulator	Fang et al., 2016
	*VvNAC08*	*Vitis vinifera*	*Arabidopsis thaliana*	Drought, SA, ABA, JA, MT inducible	Positive regulator	Ju et al., 2020a
	*VvNAC17*	*Vitis vinifera*	*Arabidopsis thaliana*	Drought, high temperature, cold, SA, ABA inducible	Positive regulator	Ju et al., 2020b
	*VaNAC17*	*Vitis amurensis*	*Arabidopsis thaliana*	PEG, MeJA, and ABA-inducible	Positive regulator	Su et al., 2020
ZFP	*PdC3H17*	*Populus deltoides*	*Populus deltoides* × *P. euramericana* “nanlin895”	Unknown	Positive regulator	Zhuang et al., 2020
bZIP	*PtabZIP1L*	*Populus tremula* × *P. alba*	*Populus tremula* × *P. alba*	PEG inducible	Positive regulator	Dash et al., 2017
WOX	*PagWOX11/12a*	*Populus alba* × *P. glandulosa* cv. “84K”	*Populus alba* × *P. glandulosa* cv. “84K”	PEG inducible	Positive regulator	Wang et al., 2019
HD-Zip	*EcHB1*	*Eucalyptus camaldulensis*	*Eucalyptus grandis* × *E. urophylla*	Unknown	Positive regulator	Sasaki et al., 2019
	*MdHB-7*	*Malus domestica*	*Malus domestica*	ABA and drought	Positive regulator	Zhao S. et al., 2020

### WRKYs

Some other TF families do not recognize the signature ABRE motif but play an important role in ABA-dependent stress response (Singh and Laxmi, 2015). As one of the largest plant TF families, the WRKY TF family features the conserved WRKY domain which shows potential DNA-binding activity. WRKYs recognize the W-box (TTGACC/T) that has been found in the promoters of many biotic and abiotic stress-responsive genes (Chen et al., 2019). WRKY18, WRKY40, and WRKY60 negatively regulate ABA-responsive genes (i.e., *ABI4*, *ABI5*, and *ABF4*) by directly binding to their promoters under normal conditions, and the *wrky40* mutant showed ABA-hypersensitivity phenotypes. Expression of *WRKY18*, *WRKY40*, and *WRKY60* are induced by water deficiency as well as ABA, and ABA also promotes the ABAR-WRKY40 interaction to relieve the negative effect of WRKY40 on *ABI5* expression (Chen et al., 2010; Shang et al., 2010). *WRKY63/ABO3* is induced by ABA treatment, and the *abo3* mutant is more sensitive to drought stress (Ren et al., 2010). WRKY46, WRKY54, and WRKY70 have also been identified to be involved in BR-regulated drought response, and the *wrky46 wrky54 wrky70* triple mutant shows more tolerance to drought stress (Chen et al., 2017). In addition to negatively regulating drought stress, WRKY40 and WRKY70 are known repressors in plant immunity, and it will be interesting to investigate their roles in the crosstalk of pathogen and drought stress, given that it has been reported that drought stress-induced ABA biosynthesis inhibits salicylic acid (SA)-mediated plant immunity (Chen et al., 2019; Gupta et al., 2020b). Other WRKY TFs, such as WRKY28 and WRKY21, were reported to positively and negatively regulate plant response to drought stress, respectively, (Babitha et al., 2013; Zhao K. X. et al., 2020; Figure 1).

Genome-wide identification of the WRKY TF family have been carried out in several woody plants, such as poplar and grapevine (He et al., 2012; Jiang et al., 2014; Wang et al., 2014). Over 100 putative *PtrWRKY* genes have been identified in *Populus*, and several WRKY members in group III showed enhanced expression in response to drought treatment. It would be interesting to further investigate the function of these drought-responsive *PtrWRKYs*, such as *PtrWRKY89*, whose expression is strongly induced by ABA and PEG treatment (He et al., 2012; Wang et al., 2015). Recently, a drought and ABA-induced *PbrWRKY53* was isolated from *Pyrus betulaefolia*. Overexpression of *PbrWRKY53* increased drought tolerance in tobacco and *Pyrus ussuriensis*, whereas the *PbrWRKY53* knock-down lines showed compromised drought tolerance in *P. ussuriensis* with reduced *PbrNCED1* and *PbrNCED3* expression levels (Liu Y. et al., 2019; Table 1). The function of WRKY TFs in biotic stress response has been well-studied in woody plants (Duan et al., 2015; Jiang et al., 2017; Liu G. et al., 2019). However, their function in abiotic stress response, especially in drought response, needs to be deeply investigated.

### NF-Ys

NF-Ys consist of three subunits (NF-YA, NF-YB, and NF-YC) and recognize the CCAAT box in the promoters of target genes. *Arabidopsis AtNF-YA5* is induced by drought stress in an ABA-dependent manner, and the mutant *nf-ya5* shows hypersensitivity to drought stress, whereas overexpression of NF-YA5 enhances drought tolerance (Li et al., 2008). Other members in NF-YA family, such as AtNF-YA3, AtNF-YA7, and AtNF-YA10 are positive regulators of drought response (Leyva-Gonzalez et al., 2012). *AtNF-YB2*, induced by dehydration stress in both ABA-dependent and ABA-independent manners, also positively regulates drought tolerance (Sato et al., 2019). It was also reported that AtNF-YC3/4/9 enhances drought-escape-responsive flowering by interacting with ABF3/4 and activates *SOC1* expression under drought stress (Hwang et al., 2019; Figure 1).

There are 52 *NF-Y* genes, including 13 *NF-YAs*, 20 *NF-YBs*, and 19 *NF-YCs* identified in the *P. trichocarpa* genome. Among 13 *PtNF-YA* genes, 11 of them were found to contain the ABRE element in their promoter regions, which correlates with their expressions being induced by abiotic stresses. Noticeably, *PtNF-YA2* and *PtNF-YA4* were induced by polyethylene glycol-simulated drought treatment (Liu et al., 2020). Although the expression of *PtNF-YA9* was not induced by dehydration, *Arabidopsis* plants overexpressing *PtNF-YA9* showed high tolerance to drought stress by promoting ABA-dependent stomatal closure in leaves (Lian et al., 2018). Similarly, PdNF-YB7 identified from *Populus nigra* × (*P. deltoides* × *P. nigra*) was also reported to increase drought tolerance in *Arabidopsis* by regulating ABA-dependent dehydration response (Han et al., 2013). Overexpression of *PdNF-YB21*, another *PdNF-YB*, enhanced root growth and drought tolerance, while the knockout mutant showed the opposite phenotypes in poplar [*Populus nigra* × (*P. deltoides* × *P. nigra*)] (Zhou et al., 2020). Further investigation showed that PdNF-YB21 interacts with a B3 domain TF PdFUSCA3 and activates the expression of ABA biosynthesis genes (Suzuki and McCarty, 2008). Accumulation of ABA in the roots of *PdNF-YB21* overexpression plants increased root growth and enhanced drought tolerance by promoting IAA transport (Zhou et al., 2020; Table 1). Collectively, these results indicate that NF-Y TFs have important functions in drought response in the ABA-dependent pathway in woody plants.

### MYBs and Other TF Families

For some drought-responsive genes, such as *RD22* and *AtADH1*, their expression is dependent on ABA. However, the ABRE *cis*-elements are not found in their promoters. Further studies identified two *cis*-elements, specifically *MYBRS* (C/TAACNA/G) and *MYCRS* (CANNTG), in their promoters. The MYB TF family member *At*MYB2 and the bHLH TF *At*MYC2 have been identified to recognize these elements, respectively, (Abe et al., 1997, 2003). AtAMYB96 is another MYB TF involved in drought response, and the *myb96-1* mutant showed susceptibility to drought, whereas transgenic plants overexpressing *AtMYB96* showed enhanced drought tolerance. In addition to the activation of target genes in response to ABA, AtMYB96 was reported to negatively regulate ABA-repressible genes *RHO GTPASE OF PLANTS* (*ROPs*) by recruiting HDA15 (Seo et al., 2009; Lee and Seo, 2019; Figure 1).

There are other TF families that regulate drought response via the ABA-dependent pathway. ABA-induced transcriptional repressors (AITRs) belong to a novel TF family, and the double mutant of *aitr2 aitr5* and triple mutant of *aitr2 aitr5 aitr6* show strong drought tolerance. Further analysis shows that these AITRs are able to target and repress key regulators in ABA signaling, such as *ABI2* (Tian et al., 2017). Auxin-sensitive Aux/IAA transcriptional repressors are also reported to be involved in drought response. The triple mutant of *iaa5 iaa6 iaa19* shows decreased drought tolerance. Further analysis indicated that IAA5/6/19, induced by drought and ABA, regulate stomatal closure by adjusting glucosinolate levels (Salehin et al., 2019).

As one of the largest TF families, there are over 190 MYB TFs identified in poplar (Wilkins et al., 2009). *PtoMYB170* was identified from *Populus tomentosa* (Chinese white poplar), and overexpression of *PtoMYB170* in *Arabidopsis* increased drought tolerance by promoting dark-induced stomatal closure (Xu et al., 2017). Overexpression of *PtrMYB94* increased drought tolerance in both poplar and *Arabidopsis*. Further analysis revealed that *PtrMYB94* enhanced drought tolerance in an ABA-dependent manner by activating ABA-responsive genes and increasing ABA content (Fang et al., 2019; Table 1). Compared with *Arabidopsis*, studies of MYB TFs in drought response in poplar are still limited.

## ABA-Independent Drought Response Pathway

Although many TFs are induced by ABA and function in the ABA-dependent signaling pathway, there are some TFs whose expression is highly induced by water deficiency, but not primarily mediated by ABA biosynthesis or signaling pathway (Yoshida et al., 2014). DREB2s and some NACs belong to this category.

### AP2/ERFs

Dehydration-responsive element DRE (A/GCCGAC) was identified in many drought-responsive genes, which is recognized by DREB2 proteins. DREB2A and DREB2B belong to the AP2/ERF (Apetala2 and ethylene-responsive factors) TF family (Maruyama et al., 2012). The expression of *DREB2A* is slightly induced by ABA, but highly induced by dehydration, supporting the ABA-independent manner in response to drought stress (Kim et al., 2011). Overexpression of the constitutively active DREB2A (DREB2A-CA) increases drought tolerance. The active form of DREB2A-CA lacks the PEST sequence that is a negative regulatory domain for DREB2A, and makes this protein unstable under non-stressful conditions (Sakuma et al., 2006). Further analysis revealed that protein stability of DREB2A is controlled by DRIP1 and DRIP2 through the ubiquitin/26S proteasome system under normal conditions (Qin et al., 2008). TINY belongs to AP2/ERF family in Arabidopsis. *tiny tiny2 tiny3* triple mutant showed hypersensitive phenotypes to drought stress whereas transgenic plants overexpressing *TINY* enhanced drought tolerance. TINY positively regulates drought-responsive genes and promotes ABA-mediated stomatal closure by interacting with BES1 in BR signaling (Xie et al., 2019). Other ERF/AP2 TF family members such as HARDY (HRD), TG/RAP2.4A, and AtERF74 were also reported to positively regulate drought tolerance, while AtRAP2.1 negatively regulates drought response (Karaba et al., 2007; Dong and Liu, 2010; Zhu et al., 2014; Yao et al., 2017; Figure 1).

In woody species, there are 202 and 149 AP2/ERFs (consisting of ERF, RAV, APW, and Soloist subfamilies) in *Populus* and *Vitis vinifera*, respectively, (Zhuang et al., 2008; Licausi et al., 2010). PeSHN1, an AP2/ERF member isolated from *Populus* × *euramericana* “Neva,” was reported to promote wax biosynthesis by targeting *PeLACS2* and other related genes. It was concluded that poplar plants overexpressing *PeSHN1* enhance drought tolerance by increasing wax biosynthesis and reducing transpiration (Meng et al., 2019). Orthologs of *Arabidopsis DREB2A*, *PeDREB2a* and *PeDREB2L* from *P. euphratica* were shown to be involved in drought response. Overexpression of *PeDREB2a* or *PeDREB2L* in *Arabidopsis* enhances drought tolerance although the potential PEST sequences were identified within the protein sequence. These results suggest that PeDREB2s are more stable than DREB2A and may function as the constitutively active form (Chen et al., 2011; Zhou et al., 2012). PagERF35, whose encoding gene is induced by drought treatment, can directly bind to the DRE motifs in the promoter of *PagWOX11/12a*. PagWOX11/12a, belonging to the WUSCHEL-related homebox (WOX) TF familiy, positively regulates drought tolerance by increasing root growth. In addition, co-expression analysis and transcriptional activation assay suggest that ERF and WOX may form a regulatory module responding to drought stress (Wang et al., 2019). MdERF38 was shown to interact with MdMYB1 to promote anthocyanin biosynthesis in response to drought stress in apple (An et al., 2020). Another two AP2/ERF TFs, MdSHINE2 and MdWRI4, were also positively involved in drought tolerance by regulating wax biosynthesis in *Arabidopsis* (Zhang Y.-L. et al., 2019; Zhang et al., 2020; Table 1).

### NACs and Other TF Families

NAM, ATAF, and CUC (NAC) TFs are encoded by the largest plant-specific NAC gene family. Many NACs induced by drought stress or ABA were reported to bind NACRS(CGTG/A) sequence in the promoters of drought stress-responsive genes (Puranik et al., 2012). Some NACs regulate drought response through the ABA-dependent pathway, while the other NACs do so through the ABA-independent pathway. *ERD1* is a dehydration-responsive gene whose expression is induced by drought stress, but not by ABA (Nakashima et al., 1997). Further analysis revealed that the *ERD1* promoter contains both NACRS (CATGTG) and zinc finger homeodomain recognition sequences (ZFHDR, CACTAAATTGTCAC). ANAC019, ANAC055, ANAC072/RD26, and ZFHD1 were reported to bind to the promoter of *ERD1*. Overexpression of *ZFHD1* or co-overexpression of *ZFHD1* and *ANACs* show induced *ERD1* expression and drought tolerance (Tran et al., 2007; Singh and Laxmi, 2015). Interestingly, ANAC072/RD26 regulates other drought-responsive genes and enhances drought tolerance in an ABA-dependent manner (Fujita et al., 2004). Recently, phosphorylation of RD26 by BIN2 was reported to be required for the activation of RD26 in drought-responsive genes (Jiang et al., 2019). JUB1 is a drought-induced NAC TF. Overexpression of *JUB1*, driven by either *CaMV35S* promoter or the *RD29A* promoter, enhances drought response, and *JUB1* was positively regulated by HD-Zip class I TF AtHB13 (Ebrahimian-Motlagh et al., 2017). SUPPRESSOR OF MORE AXILLARY GROWTH2 (MAX2)-LIKE6 (SMXL6), SMXL7, and SMXL8 belonging to SAMX1-LIKE (SMXL) family, in addition to acting as transcriptional repressors in strigolactone signaling, negatively regulate drought response. Transcriptomic and physiological evidence suggested that these three SMXL proteins regulate drought response in both the ABA-dependent and ABA-independent pathways (Wang et al., 2020; Yang T. et al., 2020; Figure 1).

There are 163 NAC genes identified in *P. trichocarpa* (Hu et al., 2010). Three drought-responsive NAC genes were identified from *P. euphratica*, including *PeNAC034*, *PeNAC036*, and *PeNAC045*. *PeNAC036* is induced, while *PeNAC034* and *PeNAC045* are repressed by drought stress. Consequently, overexpression of *PeNAC036* in *Arabidopsis* enhances drought tolerance, while overexpression of *PeNAC034* in *Arabidopsis* reduces drought tolerance. Poplar plants overexpressing *PeNAC045* also showed a drought-sensitive phenotype (Lu et al., 2018). It was proposed that *P. euphratica* may adapt to a drought environment by activating and repressing different sets of NAC genes (Lu et al., 2018). *PtrNAC006*, *PtrNAC007*, and *PtrNAC120*, regulated by PtrAREB1, were shown to be positive regulators in drought response (Li et al., 2019). In grapevine, several NAC TFs, including VvNAC26, VvNAC08, VvNAC17, and VaNAC17, were reported to enhance drought tolerance in *Arabidopsis* (Fang et al., 2016; Su et al., 2020; Ju et al., 2020a, b; Table 1). A novel CCCH zinc finger TF, PdC3H17, was found to positively regulate drought response in poplar. Overexpression of *PdC3H17* confers drought tolerance by enhancing the ROS-scavenging abilities, as well as maintaining water potential in stem (Zhuang et al., 2020). A homeodomain leucine zipper (HD-Zip) TF, EcHB1, identified from *Eucalyptus camaldulensis*, was shown to enhance drought tolerance by increasing photosynthetic efficiency, while reducing leaf area in *Eucalyptus* (Sasaki et al., 2019). MdHB-7, another HD-Zip TF from *Malus domestica*, promoted drought tolerance by enhancing ABA content, stomatal closure, and ROS detoxification (Zhao S. et al., 2020).

## Epigenetic Regulation of Drought Response

Epigenetic regulations, including DNA methylation, histone modifications, chromatin remodeling, and small RNA, contribute to abiotic stress responses (Chang et al., 2019). Increasing evidence has demonstrated the role of histone modification and DNA methylation in response to drought stress. A histone demethylase, JMJ17, demethylates H3K4 under dehydration conditions (Huang et al., 2019). The *jmj17* mutant shows high H3K4me3 levels in drought-responsive genes, such as *OST1* and *ABF3*, and enhances target gene expression and drought tolerance (Huang et al., 2019). LIKE HETEROCHROMATIN PROTEIN 1 (LHP1) belongs to the PRC1 complex, which mediates transcriptional repression of drought-related TF genes, such as *ANAC019* and *ANAC055*. The *lhp1* mutant enhances drought tolerance by promoting the expression of drought-responsive genes (Ramirez-Prado et al., 2019). HISTONE DEACETYLASE 9 (HDA9) positively regulates drought response by interacting with ABI4 and maintaining ABA homeostasis in response to drought stress (Baek et al., 2020b).

*Populus* methylome studies revealed that drought stress changed the DNA methylation level of TF coding genes and further altered their expression pattern (Liang et al., 2014). An RNA m^6^A methyltransferase coding gene from poplar, *MTA*, was shown to enhance drought tolerance by promoting trichome and root development (Lu et al., 2020). As mentioned above, several histone modification enzymes, such as AtHDA15 and PtrADA2b-PtrGCN5, were found to interact with key TFs, such as AtMYB96 and PtrAREB1-2, to regulate drought response in *Arabidopsis* and poplar (Lee and Seo, 2019; Li et al., 2019). Epigenetic regulation is also involved in stress memory by regulating gene expression, in which TFs may also participate. Using periodically combined drought and heat treatments, several stress-related memory genes were identified through tissue-specific transcriptomic profiling studies in poplar. Among them, the homologs of *Arabidopsis HOMEOBOX7* (*HB7*) were proposed as the most prominent candidates enhancing plant photosynthesis during stress recovery (Georgii et al., 2019). In conclusion, epigenetic regulation and stress memory play important roles in drought response and tolerance, but their regulatory roles have not been well defined and deserve further investigations.

## Crosstalk Between ABA-Dependent and ABA-Independent Drought Response Pathways

Plants respond to drought stress through complex regulatory networks. TFs play key roles by regulating large numbers of downstream genes, as well as interacting with other TFs. Transcriptional regulatory networks in drought response can be predicted by analyzing transcriptome data (Zhang C. et al., 2018). Crosstalks between the ABA pathway and other pathways have been revealed to regulate drought response. For example, SMXLs from SL signaling are negatively involved in drought response by regulating both SL-responsive genes and ABA-responsive genes (Yang T. et al., 2020). Accumulated evidence supports the physical interactions among TFs from ABA-dependent and ABA-independent pathways. ABA-dependent AREBs/ABFs were found to interact with DREB2A, leading to synergistic activation of the drought-responsive genes such as *RD29A* (Lee et al., 2010). AREB1 and AREB2 were also found to interact with ABA-independent ANAC096, and their interactions enhance transcriptional activities of the AREBs (Xu et al., 2013). JA-regulated MYC2 was found to interact with the ABA-responsive ABF3, supporting the crosstalk between JA signaling and ABA signaling in response to drought stress (Liu et al., 2018). BES1 from BR signaling regulates drought response by interacting with WRKY, AP2/ERF, and NAC TFs (Nolan et al., 2020). Collectively, drought response is regulated by the interplay between ABA-dependent and ABA-independent pathways (Yoshida et al., 2014).

## Complexities and Research Strategies of Drought Response in Woody Plants

Long-lived trees experience drought stress together with other biotic and abiotic stresses. High temperatures are the most common stress occurring simultaneously with drought in the field, and trees can respond differently to a single stress versus combined stresses (Zandalinas et al., 2017). Jia et al. (2017) reported that heat and drought stresses shared responsive genes in *Populus simonii*. The hierarchical genetic regulatory networks involving several TF, such as ERF1 and RD26, were formed during these combined stress treatments. It was proposed that the co-expression networks contributed to single and/or combined stress responses by regulating hormone biosynthesis and ROS production (Jia et al., 2017). Ozone (O_3_) is another phytotoxic air pollutant. Combined effects of high ozone and drought stresses were investigated in poplar through transcriptome analysis. Twelve core TFs were predicted to be master regulators in response to the combination of high O_3_ and drought stresses. Further analysis on differentially-expressed genes (DEGs) indicated that *Populus* plants respond to O_3_ and/or drought by regulating isoprene biosynthesis and the ABA-dependent pathways (Zhang J. et al., 2019). The molecular response of combined drought and low nitrogen stress has also been studied in *Populus*. RNA-seq analyses of *Populus simonii* roots in response to drought and low nitrogen stresses revealed that drought positively regulates ammonium uptake and amino acid metabolism, which, in turn, promote drought tolerance (Zhang C. et al., 2018).

The roles of different microbes and endophytes in drought tolerance have also been revealed in poplar recently. Khan et al. (2016) reported that combined application of endophytes greatly enhanced plant biomass in *Populus deltoides* × *P. nigra* clone OP-367 in response to drought. Further analyses revealed that phytohormone production of these endophytes and ROS detoxification of inoculated plants contributed to drought tolerance (Khan et al., 2016). Using an RNA-seq approach, it was found that cyclic drought treatment increased the phytobiome in taxa which could benefit the host *P. deltoides* plants in terms of disease response, ROS metabolism, and photosynthesis (Garcia et al., 2018).

Transcriptome studies, such as RNA-seq, have been used to explore the complexities of drought stress combined with other environment factors in woody plants (Estravis-Barcala et al., 2019). The advantage of transcriptome study is that it not only unravels the transcriptional regulatory networks under any stress combination but also provides detailed transcriptional events in genotype-, tissue-, and developmental stage-specific bases (Cohen et al., 2010). Transcriptomic analyses can become more effective when combined with other omics approaches, such as metabolomics, since metabolite profiling can provide additional insights by integrating diverse transcriptomic responses (Hamanishi et al., 2015). The large number of key TFs and transcriptional regulatory networks identified from RNA-seq studies can deepen our understanding of drought response and tolerance in woody plants.

## Future Perspectives

The transcriptional regulatory network of drought response was built extensively in the model plant *Arabidopsis* during the last few decades. In comparison with model plants, knowledge of drought response in woody species is still limited. Drought response varies with species and genotypes. Recently developed high-throughput omics strategies shed light on the poplar response to drought and the transcriptional regulation underlying drought tolerance. For example, genome-wide association mapping studies (GWAS) and Expression Quantitative Trait Locus (eQTL) mapping have become effective tools to identify the genetic loci underlying variation in economically important phenotypic traits and transcriptional regulation (Muchero et al., 2018; Zhang J. et al., 2018). Systems biology approaches including genomics, transcriptomics, proteomics, metabolomics, and phenomics may help facilitate the identification and functional characterization of TFs regulating drought response in woody plants (De Ollas et al., 2019). The adoption of CRISPR/Cas9 technique in trees provides the power of precisely editing the genomic loci to uncover genes in the drought response pathway (Bewg et al., 2018).

Most studies have been conducted with single-stress studies conducted in laboratory conditions. Forest trees often endure multiple stresses, such as heat and drought in field. It is imperative to verify the function of drought-related TFs and observe their impact on phenotypic expression under field conditions. The models generated from multistress studies will deepen our understanding on drought stress response and provide genetic engineering targets to create drought-tolerant and high-yield woody plants.

## Notice

This manuscript has been authored by UT-Battelle, LLC under Contract No. DE-AC05-00OR22725 with the United States Department of Energy. The United States Government retains and the publisher, by accepting the article for publication, acknowledges that the United States Government retains a non-exclusive, paid-up, irrevocable, worldwide license to publish or reproduce the published form of this manuscript, or allow others to do so, for United States Government purposes. The Department of Energy will provide public access to these results of federally sponsored research in accordance with the DOE Public Access Plan (http://energy.gov/downloads/doe-public-access-plan).

## Author Contributions

WM and J-GC conceived the study. TY drafted the manuscript. JZ, MX, GY, TT, WM, and J-GC revised the manuscript. All authors contributed to the article and approved the submitted version.

## Conflict of Interest

The authors declare that the research was conducted in the absence of any commercial or financial relationships that could be construed as a potential conflict of interest.
